# Prevalence and Correlates of Sleep Difficulties in a Large Multicenter Outpatient Oncology Population as Identified through Practice-Based Screening

**DOI:** 10.1158/2767-9764.CRC-25-0739

**Published:** 2026-03-10

**Authors:** Sarah N. Price, Laurel Zeng, Wei Sha, Dori Beeler, Kunal C. Kadakia, Arunkumar Krishnan, Declan Walsh, Kathryn E. Weaver

**Affiliations:** 1 Department of Social Sciences and Health Policy, Wake Forest University School of Medicine, Winston-Salem, North Carolina.; 2 Department of Biostatistics and Data Sciences, Atrium Health Levine Cancer Institute, Charlotte, North Carolina.; 3 Department of Cancer Medicine, Wake Forest University School of Medicine, Winston-Salem, North Carolina.; 4 Department of Supportive Oncology, Atrium Health Levine Cancer Institute, Charlotte, North Carolina.; 5 Department of Solid Tumor Oncology, Atrium Health Levine Cancer Institute, Charlotte, North Carolina.; 6 Department of Internal Medicine, Wake Forest University School of Medicine, Winston-Salem, North Carolina.; 7 Hemby Family Endowed Chair in Supportive Oncology, Atrium Health Levine Cancer Institute, Charlotte, North Carolina.; 8 Department of Implementation Science, Wake Forest University School of Medicine, Winston-Salem, North Carolina.

## Abstract

Sleep disturbances are underidentified and undertreated in oncology. To inform screening and intervention efforts at a large academic cancer center, we identified the prevalence and correlates of patient-reported sleep difficulties. This retrospective study evaluated 20,416 individuals who completed practice-based distress screening from January 2017 to June 2024. Sleep difficulties in the past 2 weeks were reported from 0 (none) to 10 (severe). Uni- and multivariable regression models evaluated the impact of sociodemographic factors (age, sex, race/ethnicity, financial concerns) and medical/psychosocial factors (cancer type/stage, time since diagnosis, distress, anxiety, depression, pain, relationship problems, tobacco and alcohol use) on clinically significant (≥5) and severe (≥7) sleep difficulties. All variables were obtained from patients’ first screen, except for cancer type, stage, and diagnosis date, which were from the cancer registry. Among the sample (59% female, 78% non-Hispanic white, 49% stage I to II, mixed cancer sites), 36% reported clinically significant sleep difficulties. In a multivariable model, the strongest correlates (OR ≥ 1.35) of sleep difficulties ≥5 were as follows: age 40 to 64 (OR = 1.35), financial concerns (OR = 1.35), distress (OR = 3.06), pain (OR = 2.80), anxiety (OR = 2.31), and depression (OR = 1.69). Risk varied by cancer type, with upper gastrointestinal, genitourinary, thoracic, and hematologic cancers showing modestly higher odds. Sensitivity analyses (cutoff ≥7, within 90 days of diagnosis) showed broadly similar but not identical correlates. Sleep difficulties are prevalent and correlated with psychosocial concerns and cancer type. Future research should explore sleep trajectories and potential care pathways for patients screening positive.

**Significance:**

This large, multicenter study demonstrates that sleep difficulties are highly prevalent among oncology outpatients and are strongly associated with psychosocial distress, pain, and cancer type. Routine screening can identify at-risk patients, supporting the need for integrated, multisymptom management strategies to improve outcomes in cancer care.

## Introduction

Sleep problems are among the most common and burdensome issues experienced by cancer survivors during and after treatment, with detrimental impacts on quality and potentially length of life (1–5), yet they are underidentified in the oncologic context. Problems initiating and maintaining sleep are frequently reported among patients’ top quality of life concerns (1, 4) and have been linked to worse mental and physical health outcomes in a variety of cancer populations (6–9). Although sleep problems are common in the general population (10%–31%), cancer-related factors (e.g., pain, treatment side effects, and disease-related stress) may initiate or exacerbate sleep problems, and oncology populations generally show higher and more variable rates of sleep issues (21%–60%; refs. 10–13). Sleep difficulties tend to cluster together with other cancer-related symptoms and psychosocial issues (e.g., fatigue, pain, anxiety, depression) and may be a key driver of global distress in cancer survivors (14–17). Evidence suggests that across cancer types and methodologies, objectively and subjectively measured sleep disturbances predict reduced survival among patients with metastatic disease although larger studies are needed (18–22). Despite their prevalence and significance, sleep problems are infrequently recognized and often unaddressed in cancer care settings (23). A survey of National Comprehensive Cancer Network (NCCN) and National Cancer Institute (NCI)–designated cancer centers found that only 56% of centers screen for sleep disorders, and no program reported that >50% of their survivors were receiving optimal insomnia-related care (24). These findings underscore the need to better identify individuals at risk for sleep problems early and to understand outcomes for those who report sleep-related issues in practice-based screening.

As sleep problems are underassessed in routine oncology care, existing prevalence estimates come primarily from surveys collected for research purposes or in the context of clinical trials (1, 4, 5, 7). These samples are often smaller and less representative of clinical practice (3, 25), which is especially limiting for comparisons by cancer type (10). Previous research using routine screening data has contributed significantly to our understanding but explored the prevalence and correlates of sleep difficulties using a smaller sample and a limited set of potential clinical and psychosocial correlates (26). Atrium Health Levine Cancer Institute, a high-volume, not-for-profit academic-community cancer center, systematically screens patients for psychosocial issues (including sleep difficulties) and has been doing so since January 2017, offering the opportunity to examine sleep difficulties among a large and diverse population in a real-world setting. Confirming the prevalence and correlates of sleep difficulties in a contemporary, diverse, and systematically screened population adds robust, generalizable data to the literature. Furthermore, these findings will facilitate the identification of individuals at risk for poor outcomes, elucidate the patterning of sleep difficulties with other cancer-related symptoms, and determine areas where greater resources are needed to address sleep problems. The present study aimed to (i) assess the prevalence of sleep difficulties based on the frequencies and percentages of patients reporting mild, moderate, severe, and potentially clinically significant sleep difficulties and (ii) evaluate the association between potentially clinically significant sleep difficulties and sociodemographic/medical factors. Based on previous research (1–5, 10, 25, 27), we hypothesized that 30% to 50% of all patients with cancer completing the Electronic Distress Screener (EDS) would report potentially clinically important sleep difficulties and that patients who are female, experiencing financial strain, anxiety, depression, and greater pain and distress would be at greatest risk.

## Materials and Methods

### Data source

This retrospective study was approved by the Wake Forest University School of Medicine institutional review board (ONC-CCS-2404; IRB00114518). Informed consent was not obtained from participants as data were collected as part of standard care, risks were considered minimal, and analyses were conducted retrospectively. Data were obtained from two separate sources: (i) electronic distress screenings (under parent protocol LCI-NOS-SUPP-TRID-001; IRB00082386) and (ii) the cancer registry at Atrium Health Levine Cancer Institute.

### Eligibility

Atrium Health Levine Cancer Institute routinely administers the EDS, with the goal of screening all ambulatory oncology patients via an electronic tablet at their initial consultation (e.g., first visit with medical oncology, radiation oncology, or surgical oncology). Therefore, most patients’ initial EDS occurs between diagnosis and treatment initiation. The EDS may be readministered at subsequent visits, but the timing and frequency of readministrations vary based on clinical workflow and patient needs, so the present analyses focus on the initial EDS to maximize sample size and analytic clarity. The EDS includes patient-reported demographic, physical, and psychosocial assessments linked to the electronic health record and contains validated triggers for automated referrals based on depression, anxiety, and malnutrition risk (28). There is currently no trigger for automatic referral based on sleep difficulties.

We conducted a retrospective analysis of patients’ initial EDS data, provided they met the following criteria: (i) age ≥18 years; (ii) completed an initial EDS from January 1, 2017, to June 28, 2024; (iii) diagnosed with cancer, any type or stage except 0, inclusive of hematologic cancers and melanoma, confirmed via matching with the cancer registry; and (iv) provided EDS data on sleep difficulties. We excluded patients who had multiple cancer sites listed in the registry and all incomplete cases (e.g., missing medical, psychosocial, or substance use variables from EDS). We chose to exclude patients with more than one primary cancer diagnosis to reduce heterogeneity and ensure the interpretability of analyses examining difficulties by cancer type.

### Study variables

Variables from the EDS included sociodemographic and psychosocial factors, symptoms, and substance use, whereas tumor type, stage, and date of diagnosis were derived from the tumor registry.

Sociodemographic factors were patient-reported in the EDS and included age at initial assessment (18–39, 40–64, 65–79, 80+), sex (female/male), and race/ethnicity [non-Hispanic White, Black or African American, Asian, American Indian or Alaska Native, Native Hawaiian or Other Pacific Islander, or Hispanic/Latino (any race)]. Age categories were created to reflect clinically meaningful life stages frequently represented in oncology research, including research into the needs of older adults (29). Participants completed a self-report item labeled “gender” with binary response options (male/female). Given the constrained response format, this variable is treated as a binary sex variable in analyses.

Psychosocial factors included insurance or financial problems/concerns, which were derived from the EDS question: “Do you have insurance/financial problems or concerns?” with options yes or no, and problems with personal relationships, which were derived from the EDS question: “Are you having problems with personal relationships?” with options no, a little, or a lot [recoded into yes (a little/a lot) vs. no].

Symptoms (including sleep difficulties, distress, and pain) are evaluated through the EDS using a single-item 11-point numeric rating scale modeled after the Edmonton Symptom Assessment System (30). Patients rated the severity of their symptoms over the past 2 weeks from 0 (none) to 10 (severe or disabling). For objective 1, the prevalence of sleep difficulties was defined by describing the numbers and percentages of patients reporting each level of sleep difficulty severity: none (0), mild (1–3), moderate (4–6), and severe (7–10; ref. 31), as well as the number and percentage of patients endorsing sleep difficulties meeting the potentially clinically important/significant threshold (≥5; ref. 32). This threshold was selected based on prior psychometric studies and distributional characteristics (32). Pain and distress were dichotomized into none/mild (0–3) versus moderate/severe (4–10). Anxiety and depression were evaluated through the Generalized Anxiety Disorder-2 item (GAD-2) and Patient Health Questionnaire-2 item (PHQ-2), respectively, with scores of ≥3 considered a positive screen for both concerns, indicating a potential clinical issue warranting further evaluation (33, 34).

Substance use included two EDS questions: (i) “In the past 30 days, how many days did you smoke cigarettes, cigars, pipes, or use smokeless tobacco?” to which respondents indicated a number of days ranging from 0 to 30 and (ii) “How often do you have a drink containing alcohol?” with response options never, less than monthly, monthly, weekly, 2 to 3 times a week, 4 to 6 times a week, or daily. Tobacco use was recoded as yes (≥1 day) or no (0 days), and alcohol use was recoded as 4 to 6 times a week to daily, 2 to 3 times a week to monthly, and never.

Consistent with previous EDS research (35), cancer variables were derived by merging EDS data with the Levine Cancer Institute cancer registry. Cases were recoded into the following cancer types: breast, upper gastrointestinal (GI), lower GI, gynecologic, head and neck, genitourinary, thoracic, hematologic, and other. Stage was recoded into I to II, III to IV, and not defined or unknown. Time since diagnosis (in days) was operationalized by calculating the time between the patient’s diagnosis (from the registry) and the patient’s initial EDS screen date (recoded into <24 days vs. ≥24 days based on the median time since diagnosis in the sample).

### Statistical methods

For each patient, a binary variable indicated whether the patient had potentially clinically significant sleeping difficulties (<5 vs. ≥ 5). Corresponding 95% confidence intervals were estimated using the Clopper–Pearson method. Patient characteristics were described using either median (range) or frequency (percent). We used uni- and multivariable logistic regression to evaluate the impact of sociodemographic factors (age, sex, race/ethnicity) and medical factors, including symptoms and psychosocial issues (pain, distress, depression, anxiety, problems with relationships, financial concerns), substance use (tobacco, alcohol), and cancer type, stage, and time since diagnosis on sleep difficulties. The correlation matrix was used to detect multicollinearity. High correlations were identified if correlation coefficients were close to 1 or −1. All observed correlation coefficients were below 0.45 (between cancer site and gender). Variance inflation factors (VIF) were calculated for all predictors. Variables were highly correlated if VIFs were greater than 5; all variables were below this threshold. Sensitivity analyses examined whether the pattern of predictors identified for moderate-to-severe sleep disturbance (≥5) remained consistent when applying a more stringent threshold for severe disturbance (≥7). We also conducted sensitivity analyses limiting the study population to those within 90 days of their cancer diagnosis to examine the consistency of results. We did not account for multiplicity, as the analyses were exploratory. Results should therefore be interpreted with caution given potential type I error inflation. All analyses used complete cases. All statistical tests were two-sided, and a *P* value < 0.05 was considered statistically significant. Statistical analysis was conducted using SAS version 9.4.

## Results

### Study population and prevalence of sleep difficulties

We matched 27,014 adults who completed an initial EDS from January 1, 2017, to June 28, 2024, with an entry in the cancer registry. Of these adults, 3,691 (13.7%) were excluded for having stage 0 disease, 1,327 (4.9%) were excluded for having more than one diagnosis, and an additional 1,580 (5.8%) were excluded for having incomplete EDS data, resulting in a final sample of 20,416. Most patients in the sample (15,023, 73.6%) completed their EDS within (±) 90 days of their cancer diagnosis. The median time since diagnosis was 24 days. Of the final sample of 20,416, only 6,874 (33.7%) reported no difficulty sleeping at all; 4,918 (24.1%) reported mild, 4,647 (22.8%) reported moderate, and 3,977 (19.5%) reported severe sleep difficulties. A total of 7,352 (36%) reported sleep difficulties at or exceeding the threshold of potential clinical importance (≥5).

A description of the study population is provided in Table 1. Briefly, among 20,416 patients, 12,075 (59.1%) identified as female, 15,855 (77.7%) as non-Hispanic white, and 10,440 (51.1%) were 65 years or older. Common cancer types included breast (5,089, 24.9%), genitourinary (2,847, 13.9%), and thoracic (2,338, 11.5%).

**Table 1.** Characteristics of adult patients with cancer completing electronic distress screening (*N* = 20,416).

Characteristic	*N* (%)
Clinically important sleep difficulty	​
No (<5)	13,064 (64)
Yes (≥5)	7,352 (36)
Sleep difficulty severity	​
None (0)	6,874 (33.7)
Mild (1–3)	4,918 (24.1)
Moderate (4–6)	4,647 (22.8)
Severe (7–10)	3,977 (19.5)
Sex	​
Female	12,075 (59.1)
Male	8,341 (40.9)
Age at initial assessment	​
18–39	1,027 (5)
40–64	8,949 (43.8)
65–79	8,477 (41.5)
80+	1,963 (9.6)
Race/ethnicity	​
Non-Hispanic White	15,855 (77.7)
Non-Hispanic Black or African American	3,642 (17.8)
Asian	338 (1.7)
American Indian or Alaska Native	87 (0.4)
Native Hawaiian or Other Pacific Islander	12 (0.1)
Hispanic/Latino (any race)	482 (2.4)
Insurance or financial problems/concerns	​
Yes	4,315 (21.1)
No	16,101 (78.9)
Problems with personal relationships	​
Yes (a little/a lot)	1,938 (9.5)
No	18,478 (90.5)
Pain in the past 2 weeks	​
None/mild (0–3)	12,616 (61.8)
Moderate/severe (4–10)	7,800 (38.2)
Distress in the past 2 weeks	​
None/mild (0–3)	8,505 (41.7)
Moderate/severe (4–10)	11,911 (58.3)
Anxiety (GAD-2)	​
No (<3)	17,204 (80.6)
Yes (≥3)	4,148 (19.4)
Depression (PHQ-2)	​
No (<3)	16,449 (80.6)
Yes (≥3)	3,967 (19.4)
Tobacco use in the past 30 days	​
No	16,853 (82.5)
Yes	3,563 (17.5)
Alcohol status	​
4–6 times a week to daily	1,864 (9.1)
2–3 times a week to monthly	8,363 (41)
Never	10,189 (49.9)
Cancer type	​
Breast	5,089 (24.9)
Genitourinary	2,847 (13.9)
Gynecologic	1,536 (7.5)
Head and neck	1,114 (5.5)
Upper GI	1,602 (7.8)
Lower GI	1,297 (6.4)
Thoracic	2,338 (11.5)
Hematologic	2,209 (10.8)
Other	2,384 (11.7)
Cancer stage	​
I–II	10,040 (49.2)
III–IV	6,162 (30.2)
Unknown or not defined	4,214 (20.6)
Time since diagnosis in days (median, range)	24, −2,677 to 25,244

### Univariable analyses

In univariable analyses, female sex, younger age, race/ethnicity, insurance or financial problems/concerns, problems with personal relationships, pain, distress, anxiety, depression, tobacco use, alcohol use, cancer type, and cancer stage were all associated with clinically meaningful sleep difficulties (*P*s > 0.001; Table 2).

**Table 2.** Correlates of potentially clinically significant sleep difficulties (≥5/10) among adult patients with cancer in uni- and multivariable logistic regression models (*N* = 20,416).

​Characteristic	Clinically significant sleep difficulty*N* (%)	Univariable analysis			Multivariable analysis		
		OR	95% CI	*P* value	OR	95% CI	*P* value
Sex	​	​	​	​	​	​	​
Female	4,583 (38)	1.23	1.16–1.31	<0.001	1.18	1.08–1.28	<0.001
Male	2,769 (33.2)	—	—	—	—	—	—
Age at initial assessment	​	​	​	​	​	​	​
18–39	414 (40.3)	1.45	1.24–1.70	<0.001	1.24	1.03–1.50	0.022
40–64	3,576 (40)	1.43	1.29–1.58	<0.001	1.36	1.20–1.54	<0.001
65–79	2,738 (32.3)	1.02	0.92–1.14	0.662	1.11	0.98–1.25	0.094
80+	624 (31.8)	—	—	—	—	—	—
Race/ethnicity	​	​	​	​	​	​	​
Non-Hispanic White	5,541 (34.9)	—	—	—	—	—	—
Non-Hispanic Black/African American	1,459 (40.1)	1.24	1.16–1.34	<0.001	0.99	0.91–1.08	0.811
Asian	107 (31.7)	0.86	0.68–1.09	0.209	0.92	0.71–1.2	0.529
American Indian or Alaska Native	39 (44.8)	1.51	0.99–2.31	0.056	1.11	0.66–1.85	0.701
Native Hawaiian or Other Pacific Islander	4 (33.3)	0.93	0.28–3.09	0.907	0.51	0.13–1.95	0.324
Hispanic or Latino (any race)	202 (41.9)	1.34	1.12–1.61	0.002	1.07	0.87–1.32	0.518
Insurance or financial concerns	​	​	​	​	​	​	​
No	5,092 (31.6)	—	—	—	—	—	—
Yes	2,260 (52.4)	2.38	2.22–2.55	<0.001	1.35	1.24–1.47	<0.001
Problems with personal relationships	​	​	​	​	​	​	​
Yes (a little/a lot)	1,121 (57.8)	2.70	2.45–2.97	<0.001	1.31	1.17–1.47	<0.001
No	6,231 (33.7)	—	—	—	—	—	—
Pain in the past 2 weeks	​	​	​	​	​	​	​
None/mild (0–3)	2,714 (21.5)	—	—	​	—	—	​
Moderate/severe (4–10)	4,638 (59.5)	5.35	5.03–5.69	<0.001	2.78	2.59–2.99	<0.001
Distress in the past 2 weeks	​	​	​	​	​	​	​
None/mild (0–3)	1,200 (14.1)	—	—	​	—	—	​
Moderate/severe (4–10)	6,152 (51.6)	6.50	6.06–6.98	<0.001	3.06	2.82–3.31	<0.001
Anxiety (GAD-2)	​	​	​	​	​	​	​
None/mild (<3)	4,606 (28)	—	—	​	—	—	​
Moderate/severe (≥3)	2,746 (69.2)	5.78	5.36–6.24	<0.001	2.31	2.1–2.54	<0.001
Depression (PHQ-2)	​	​	​	​	​	​	​
None/mild (<3)	5,143 (29.8)	—	—	​	—	—	​
Moderate/severe (≥3)	2,209 (70.6)	5.67	5.22–6.16	<0.001	1.68	1.51–1.87	<0.001
Tobacco use in the past 30 days	​	​	​	​	​	​	​
No	5,636 (33.4)	—	—	​	—	—	—
Yes	1,716 (48.2)	1.85	1.72–1.97	<0.001	1.11	1.02–1.22	0.02
Alcohol status	​	​	​	​	​	​	​
4–6 times a week to daily	526 (28.2)	—	—	—	—	—	—
2–3 times a week to monthly	2,849 (34.1)	1.31	1.18–1.47	<0.001	1.13	0.99–1.28	0.066
Never	3,977 (39)	1.63	1.46–1.81	<0.001	1.16	1.02–1.31	0.027
Cancer type	​	​	​	​	​	​	​
Breast	1,636 (32.1)	—	—	—	—	—	—
Genitourinary	875 (30.7)	0.94	0.85–1.03	0.194	1.22	1.05–1.38	0.009
Gynecologic	603 (39.3)	1.36	1.21–1.54	<0.001	1.00	0.87–1.15	0.968
Head and neck	405 (36.4)	1.21	1.05–1.38	0.007	1.00	0.84–1.18	0.979
Upper GI	754 (47.1)	1.88	1.67–2.10	<0.001	1.28	1.10–1.48	0.001
Lower GI	481 (37.1)	1.24	1.10–1.41	<0.001	1.09	0.93–1.28	0.267
Thoracic	1,042 (44.6)	1.70	1.53–1.88	<0.001	1.18	1.04–1.35	0.011
Hematologic	825 (37.3)	1.26	1.13–1.40	<0.001	1.14	1.00–1.32	0.057
Other	731 (30.7)	0.93	0.84–1.03	0.198	0.96	0.84–1.10	0.535
Cancer stage	​	​	​	​	​	​	​
I–II	3,221 (32.1)	—	—	—	—	—	—
III–IV	2,609 (42.3)	1.55	1.46–1.66	<0.001	1.05	0.97–1.15	0.221
Unknown or not defined	1,522 (36.1)	1.20	1.11–1.29	<0.001	1.05	0.96–1.16	0.293
Time since diagnosis	​	​	​	​	​	​	​
<24 days	3,924 (39)	1.3	1.22–1.37	<0.001	1.08	1.00–1.15	0.041
≥24 days	3,428 (33.1)	—	—	—	—	—	—

Although non-Hispanic Black or African American race and Hispanic or Latino ethnicity were associated with a greater risk for sleep difficulties in univariable analyses compared with non-Hispanic white race/ethnicity (Table 2), this difference was no longer significant in multivariable analyses, and race/ethnicity was excluded from the final model. Unknown or not defined stage and stage III to IV (vs. stage I–II) were also associated with increased risk for sleep difficulties in a univariable model, but not in a multivariable model; cancer stage was also ultimately excluded from the final multivariable model. Head and neck and lower GI cancer and alcohol consumption 2 to 3 times per week to monthly were also significantly associated with sleep difficulties in univariable models but were no longer significantly associated in the multivariable model (although alcohol use and cancer type were retained; see Table 2). Variables retained in the final multivariable model were sex, insurance or financial problems/concerns, problems with personal relationships, pain, distress, depression, anxiety, tobacco use, alcohol use, time since diagnosis, and cancer type.

### Multivariable analyses

**Figure 1.**
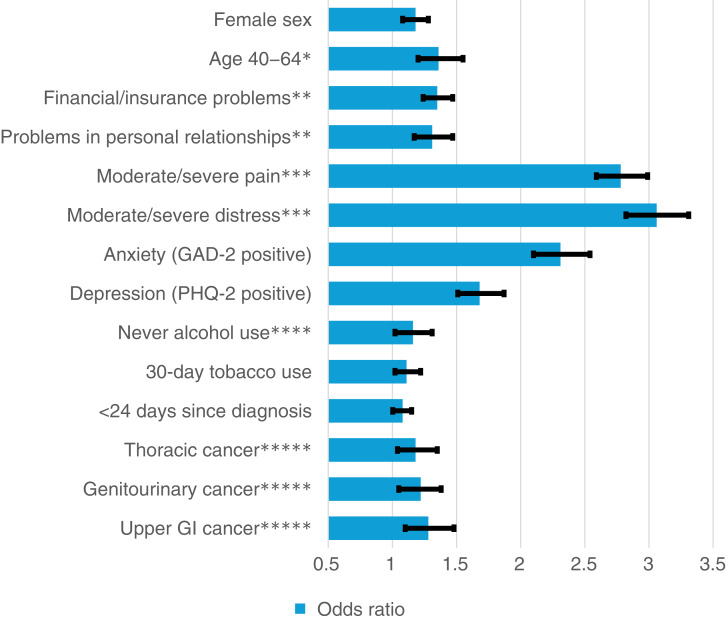
Characteristics independently associated with greater odds of clinically significant sleep difficulties (≥5/10) among adult patients with cancer (*N* = 20,416) in a multivariable logistic regression model. *, age 80+; **, none; ***, none/mild; ****, 4–6 times a week or more; *****, breast.

In the multivariable model, female sex [vs. male; OR = 1.18 (1.08–1.28), *P* < 0.001], age group 40 to 64 [vs. 80+; OR = 1.36 (1.2–1.54), *P* < 0.001], insurance or financial problems/concerns [vs. none; OR = 1.35 (1.25–1.47), *P* < 0.001], problems with personal relationships [vs. none; OR = 1.31 (1.17–1.47), *P* < 0.001], moderate/severe pain [vs. none/mild; OR = 2.78 (2.59–2.99), *P* < 0.001], moderate/severe distress [vs. none/mild; OR = 3.06 (2.82–3.31), *P* < 0.001], anxiety [GAD-2 positive; OR = 2.31 (2.1–2.54), *P* < 0.001], depression [PHQ-2 positive; OR = 1.68 (1.51–1.87), *P* < 0.001], tobacco use in the past 30 days [vs. none; OR = 1.11 (1.02–1.22), *P* = 0.02], never alcohol use [vs. 4–6 drinks per week or more; OR = 1.16 (1.02–1.31), *P* = 0.027], <24 days since diagnosis [vs. >24 days since diagnosis; OR = 1.08 (1.003–1.15), *P* = 0.041], and genitourinary [vs. breast; OR = 1.22 (1.05–1.38), *P* = 0.009], upper GI [vs. breast; OR = 1.28 (1.10–1.48), *P* = 0.001], and thoracic cancer [vs. breast; OR = 1.18 (1.04–1.35), *P* = 0.01] remained associated with increased risk for sleep difficulties (Table 2; Fig. 1).

### Sensitivity analyses

In sensitivity analyses using a higher threshold for sleep disturbance (≥7; severe; Table 3), the overall pattern of associations between patient characteristics and sleep difficulties remained largely consistent with those observed using the clinically significant cutoff (≥5; Table 2). Higher psychosocial symptom burden and younger age continued to correlate with increased risk. However, several differences emerged in the multivariable models. Specifically, sex, time since diagnosis, and genitourinary and thoracic cancer types, which were significant predictors in the main analysis (≥5) and in univariable analyses for severe sleep disturbance, were not significant independent predictors in the multivariable model for the ≥7 threshold. Additionally, age 18 to 39 [vs. 80+; OR = 1.39 (1.12–1.73), *P* = 0.003], non-Hispanic Black/African American race [vs. non-Hispanic White; OR = 1.14 (1.03–1.26), *P* = 0.013], and stage III to IV disease [vs. I–II; OR = 1.12 (1.02–1.23), *P* = 0.02] were associated with a greater risk of severe sleep disturbance in the multivariable model, whereas these associations were not significant in the main model.

**Table 3.** Sensitivity analyses: correlates of severe sleep difficulties (≥7/10) among adult patients with cancer in uni- and multivariable logistic regression models (*N* = 20,416).

​Characteristic	Severe sleep difficulty *N* (%)	Univariable analysis			Multivariable Analysis		
		OR	95% CI	*P* value	OR	95% CI	*P* value
Sex	​	​	​	​	​	​	​
Female	2,429 (20.1)	1.17	1.03–1.19	0.006	1.04	0.94–1.15	0.413
Male	1,548 (18.6)	—	—	—	—	—	—
Age at initial assessment	​	​	​	​	​	​	​
18–39	240 (23.4)	1.65	1.37–1.99	<0.001	1.39	1.12–1.73	0.003
40–64	2,027 (22.7)	1.59	1.39–1.81	<0.001	1.47	1.27–1.71	<0.001
65–79	1,404 (16.6)	1.07	0.94–1.23	0.294	1.16	1.00–1.34	0.058
80+	306 (15.6)	—	—	—	—	—	—
Race/ethnicity	​	​	​	​	​	​	​
Non-Hispanic White	2,922 (18.4)	—	—	—	—	—	—
Non-Hispanic Black/African American	861 (23.6)	1.37	1.26–1.49	<0.001	1.14	1.03–1.26	0.013
Asian	57 (16.9)	0.90	0.67–1.20	0.463	1.02	0.74–1.40	0.923
American Indian or Alaska Native	22 (25.3)	1.50	0.92–2.43	0.102	1.03	0.58–1.80	0.931
Native Hawaiian or Other Pacific Islander	3 (25)	1.48	0.40–5.45	0.560	1.01	0.24–4.20	0.987
Hispanic or Latino (any race)	112 (23.2)	1.34	1.08–1.66	0.008	1.12	0.88–1.43	0.360
Insurance or financial concerns	​	​	​	​	​	​	​
No	2,604 (16.2)	—	—	—	—	—	​
Yes	1,373 (31.8)	2.42	2.24–2.61	<0.001	1.29	1.17–1.41	<0.001
Problems with personal relationships	​	​	​	​	​	​	​
Yes (a little/a lot)	697 (36)	2.60	2.35–2.88	<0.001	1.14	1.01–1.29	0.030
No	3,280 (17.8)	—	—	—	—	—	​
Pain in the past 2 weeks	​	​	​	​	​	​	​
None/mild (0–3)	1,151 (9.1)	—	—	​	—	—	​
Moderate/severe (4–10)	2,826 (36.2)	5.66	5.24–6.11	<0.001	2.72	2.48–2.97	<0.001
Distress in the past 2 weeks	​	​	​	​	​	​	​
None/mild (0–3)	458 (5.4)	—	—	​	—	—	​
Moderate/severe (4–10)	3,519 (29.5)	7.37	6.65–8.16	<0.001	2.99	2.67–3.35	<0.001
Anxiety (GAD-2)	​	​	​	​	​	​	​
None/mild (<3)	2,110 (12.8)	—	—	​	—	—	​
Moderate/severe (≥3)	1,867 (47.1)	6.04	5.59–6.53	<0.001	2.37	2.15–2.61	<0.001
Depression (PHQ-2)	​	​	​	​	​	​	​
None/mild (<3)	2,412 (14)	—	—	​	—	—	​
Moderate/severe (≥3)	1,565 (50)	6.17	5.68–6.70	<0.001	1.93	1.73–2.14	<0.001
Tobacco use in the past 30 days	​	​	​	​	​	​	​
No	2,911 (17.3)	—	—	​	—	—	​
Yes	1,066 (29.9)	2.04	1.88–2.22	<0.001	1.16	1.05–1.29	0.003
Alcohol status	​	​	​	​	​	​	​
4–6 times a week to daily	258 (13.8)	—	—	—	—	—	—
2–3 times a week to monthly	1,481 (17.7)	1.34	1.16–1.55	<0.001	1.13	0.96–1.33	0.129
Never	2,238 (22)	1.75	1.52–2.01	<0.001	1.20	1.03–1.41	0.023
Cancer type	​	​	​	​	​	​	​
Breast	787 (15.5)	—	—	—	—	—	—
Genitourinary	447 (15.7)	1.02	0.90–1.16	0.781	1.11	0.94–1.31	0.222
Gynecologic	328 (21.4)	1.48	1.29–1.71	<0.001	1.04	0.88–1.23	0.624
Head and neck	233 (20.9)	1.45	1.23–1.70	<0.001	1.08	0.89–1.32	0.434
Upper GI	453 (28.3)	2.16	1.89–2.46	<0.001	1.28	1.08–1.51	0.004
Lower GI	278 (21.4)	1.49	1.28–1.74	<0.001	1.17	0.97–1.40	0.105
Thoracic	597 (25.5)	1.87	1.66–2.11	<0.001	1.15	0.99–1.34	0.077
Hematologic	444 (20.1)	1.38	1.21–1.56	<0.001	1.15	0.97–1.35	0.106
Other	410 (17.2)	1.14	1–1.29	0.057	1.12	0.95–1.31	0.175
Cancer stage	​	​	​	​	​	​	​
I–II	1,627 (16.2)	—	—	—	—	—	—
III–IV	1,536 (24.9)	1.72	1.59–1.86	<0.001	1.12	1.02–1.23	0.022
Unknown or not defined	814 (19.3)	1.24	1.13–1.36	<0.001	1.03	0.92–1.16	0.611
Time since diagnosis	​	​	​	​	​	​	​
<24 days	2,161 (21.5)	1.29	1.20–1.38	<0.001	1.08	1.00–1.17	0.059
≥24 days	1,816 (17.5)	—	—	—	—	—	—

Similarly, when analyses were restricted to patients who completed distress screening within ± 90 days of diagnosis (Table 4), results were again broadly consistent with the main analysis. However, tobacco use was not a significant predictor in the multivariable model. Additionally, alcohol use 2 to 3 times per week to monthly [vs. never; OR = 1.21 (1.02–1.23), *P* = 0.02], stage III to IV cancer [vs. stage I–II; OR = 1.12 (1.02–1.23), *P* = 0.024], and hematologic cancer type [vs. breast; OR = 1.23 (1.04–1.44)] were associated with increased odds of sleep disturbance in this restricted sample, whereas they were not significant in the main analysis.

**Table 4.** Sensitivity analyses: correlates of potentially clinically significant sleep difficulties (≥5/10) among adult patients with cancer in uni- and multivariable logistic regression models, limited to patients ± 90 days from diagnosis (*N* = 15,023).

​Characteristic	Sleep difficulty*n* (%)	Univariable analysis			Multivariable analysis		
		OR	95% CI	*P* value	OR	95% CI	*P* value
Sex	​	​	​	​	​	​	​
Female	3,513 (38)	1.14	1.06–1.22	<0.001	1.15	1.04–1.27	0.006
Male	2,019 (35)	—	—	—	—	—	—
Age at initial assessment	​	​	​	​	​	​	​
18–39	326 (41)	1.38	1.15–1.65	<0.001	1.18	0.95–1.46	0.127
40–64	2,722 (40.5)	1.35	1.2–1.52	<0.001	1.33	1.15–1.53	<0.001
65–79	1,998 (33)	0.98	0.87–1.10	0.709	1.05	0.91–1.21	0.475
80+	486 (33.5)	—	—	—	—	—	—
Race and ethnicity	​	​	​	​	​	​	​
Non-Hispanic White	4,240 (35.8)	—	—	—	—	—	—
Non-Hispanic Black or African American	1,021 (41)	1.25	1.14–1.36	<0.001	0.96	0.86–1.06	0.394
Asian	85 (32)	0.84	0.65–1.09	0.197	0.88	0.65–1.18	0.395
American Indian or Alaska Native	28 (48.3)	1.67	1–2.81	0.05	1.05	0.56–1.97	0.878
Native Hawaiian or Other Pacific Islander	3 (30)	0.77	0.2–2.98	0.703	0.34	0.08–1.44	0.142
Hispanic or Latino (any race)	155 (43.8)	1.4	1.13–1.73	0.002	1.15	0.90–1.47	0.258
Insurance or financial problems/concerns	​	​	​	​	​	​	​
No	3,845 (32.6)	—	—	​	—	—	—
Yes	1,687 (52.5)	2.28	2.11–2.47	<0.001	1.33	1.21–1.47	<0.001
Problems with personal relationships	​	​	​	​	​	​	​
No	4,765 (34.7)	—	—	—	1.32	1.15–1.51	<0.001
Yes (a little/a lot)	767 (59.3)	2.74	2.44–3.07	<0.001	—	—	—
Pain in the past 2 weeks	​	​	​	​	​	​	​
None/mild	2,072 (22.4)	—	—	​	—	—	​
Moderate/severe	3,460 (60)	5.20	4.84–5.59	<0.001	2.70	2.48–2.94	<0.001
Distress in the past 2 weeks	​	​	​	​	​	​	​
None/mild	795 (13.7)	—	—	​	—	—	​
Moderate/severe	4,737 (51.3)	6.61	6.07–7.20	<0.001	3.10	2.82–3.41	<0.001
Anxiety (GAD-2)	​	​	​	​	​	​	​
No (<3)	3,361 (28.3)	—	—	​	—	—	​
Yes (3+)	2,171 (69.3)	5.74	5.27–6.25	<0.001	2.42	2.18–2.69	<0.001
Depression (PHQ-2)	​	​	​	​	​	​	​
No (<3)	3,847 (30.4)	—	—	​	—	—	​
Yes (3+)	1,685 (71.4)	5.73	5.20–6.31	<0.001	1.71	1.51–1.93	<0.001
Tobacco use in the past 30 days	​	​	​	​	​	​	​
No	4,212 (34.2)	—	—	​	—	—	—
Yes	1,320 (48.7)	1.82	1.68–1.98	<0.001	1.07	0.96–1.19	0.207
Alcohol Status	​	​	​	​	​	​	​
Daily and 4–6 times a week	400 (28.5)	—	—	—	—	—	—
2–3 times a week to monthly	2,182 (35.2)	1.36	1.20–1.55	<0.001	1.21	1.05–1.41	0.01
Never	2,950 (39.8)	1.66	1.46–1.88	<0.001	1.21	1.04–1.40	0.012
Cancer type	​	​	​	​	​	​	​
Breast	1,242 (31.5)	—	—	—	—	—	—
Genitourinary	486 (32.6)	1.05	0.92–1.19	0.464	1.19	0.99–1.41	0.051
Gynecological	513 (39.1)	1.40	1.23–1.59	<0.001	1.02	0.88–1.19	0.761
Head and neck	316 (37.1)	1.28	1.10–1.50	0.002	1.05	0.87–1.28	0.596
Upper GI	656 (51.5)	2.05	1.80–2.33	<0.001	1.29	1.09–1.53	0.003
Lower GI	611 (62.3)	1.32	1.14–1.52	<0.001	1.16	0.96–1.39	0.124
Thoracic	963 (53.9)	1.86	1.66–2.09	<0.001	1.26	1.08–1.47	0.003
Hematologic	947 (60.6)	1.41	1.25–1.60	<0.001	1.23	1.04–1.44	0.013
Other	1,275 (70)	0.93	0.83–1.05	0.255	0.97	0.83–1.13	0.663
Cancer stage	​	​	​	​	​	​	​
I–II	2,361 (31.5)	—	—	—	—	—	—
III–IV	2,147 (45.2)	1.79	1.66–1.93	<0.001	1.12	1.02–1.23	0.023
Unknown or not defined	1,024 (36.7)	1.26	1.15–1.38	<0.001	1.05	0.94–1.18	0.381
Time since diagnosis	​	​	​	​	​	​	​
<21	3,044 (40.8)	1.41	1.32–1.51	<0.001	1.14	1.05–1.23	0.002
≥21	2,488 (32.9)	—	—	—	—	—	—

## Discussion

This large multicenter retrospective study highlights the substantial burden of sleep difficulties among oncology outpatients, with more than one third (36%) reporting clinically significant symptoms and nearly 20% reporting sleep difficulties in the severe range. These findings reinforce prior evidence that sleep disturbances are common in cancer populations and extend existing work by leveraging a substantially larger, multicenter sample and including additional clinical and psychosocial variables (26). By using practice-based distress screening data from more than 20,000 patients, our study provides a robust characterization of sleep difficulties and their correlates across diverse cancer types and sociodemographic groups.

Consistent with prior research (7, 14, 26, 36, 37), symptoms and psychosocial factors (distress, anxiety, depression, pain, relationship problems, and financial concerns) were the most robust correlates of sleep difficulties in this population. Notably, distress (OR = 3.06) and pain (OR = 2.78) were the two strongest predictors in uni- and multivariable models, underscoring the interconnected nature of cancer-related symptoms. These findings support integrated screening and treatment approaches that consider sleep as part of a broader constellation of psychosocial and physical symptoms. Indeed, sleep disturbances often cluster together with other symptoms that are prevalent across multiple cancer types and stages (e.g., pain, fatigue, psychologic distress; ref. 38). Coordinated multisymptom management approaches may be an efficient alternative to addressing each symptom separately although more work is needed to move from single- to multisymptom management strategies, including the identification of cluster “driving” symptoms, application of symptom cluster frameworks to intervention studies, and development of decision support tools to guide the selection of overlapping management strategies (16, 39). Financial concerns and recent cancer diagnosis (<24 days) were also independent predictors, suggesting that socioeconomic vulnerability and the acute stressors associated with initial diagnosis and treatment may exacerbate sleep problems. Future research should explore how financial stressors influence sleep over time and whether addressing health-related social needs can improve sleep and symptom burden.

Sociodemographic factors were also associated with sleep difficulties, with ages 40 to 64 being the strongest sociodemographic correlate. In the main analyses, patients in the 40 to 64-year-old age group who identified as female were more likely to report sleep difficulties, aligning with general population trends and prior oncology studies. Although general older adult populations (age ≥65) tend to have high rates of sleep disturbance (40), within oncology populations, younger adults often experience greater disruptions to mood and sleep, and these changes may be especially pronounced for those below 45 (19, 25–27, 41, 42).

Cancer type was also associated with sleep difficulties, with patients diagnosed with genitourinary, upper GI, and thoracic cancers experiencing modestly higher odds compared with those with breast cancer. The lower rate of sleep difficulties among patients with breast cancer was surprising as previous population-based studies found a high rate of sleep problems (especially insomnia) among patients with breast cancer, and this group is frequently studied in behavioral sleep intervention research (43). The differences we found in the prevalence of self-reported sleep difficulties by cancer type may reflect variations in symptom burden, treatment side effects, or disease-related stress across cancer types and warrant further investigation. For example, certain sleep-disrupting issues (e.g., sleep apnea) may be more prevalent among patients with malignancies affecting the airway (e.g., head and neck, thoracic; refs. 44, 45). This is an important consideration for follow-up assessment as sleep apnea is highly comorbid with insomnia but necessitates additional management strategies (46). Future studies using multidimensional sleep assessments are needed to better characterize the nature and drivers of sleep problems by cancer type.

Alcohol and tobacco use were modestly associated with sleep difficulties. The association between never alcohol use and higher odds of sleep difficulties is unexpected, given the established sleep-disrupting effects of alcohol; however, similar findings have previously been reported and may reflect reverse causality or confounding (47–49). Although tobacco use was positively associated with sleep difficulties, in alignment with research demonstrating that nicotine can interfere with sleep, tobacco use may also be a marker of broader distress rather than a direct contributor.

Although the main analysis identified female sex, younger age, financial concerns, problems with personal relationships, pain, distress, anxiety, depression, tobacco use, alcohol use, time since diagnosis, and certain cancer types as significant correlates of sleep difficulties, sensitivity analyses mostly supported these associations with some subtle differences. When using a higher threshold for severe sleep disturbance (≥7), female sex, time since diagnosis, and genitourinary and thoracic cancer types were no longer independent predictors in the multivariable model, and age 18 to 39, non-Hispanic Black/African American race, and stage III to IV disease were significant correlates. In analyses restricted to patients screened within ± 90 days of diagnosis, stage III to IV cancer and hematologic cancer were also associated with increased risk, whereas tobacco use was not a significant predictor. These findings suggest that although physical and psychosocial symptoms are robust predictors, the associations with demographic and clinical factors may vary depending on the definition of sleep disturbance and the timing of assessment.

Although Atrium Health Levine Cancer Institute does not currently have automated referrals tied to the sleep difficulties question, the results of this study can inform such processes for this and other centers. Although a threshold of ≥5 was selected to define sleep difficulties in these analyses based on previous psychometric work and distributional characteristics, exploring alternative cut points to define a positive screen could help optimize sensitivity and specificity for identifying patients in need of follow-up. This is particularly relevant in settings where resource constraints necessitate targeted referrals (28). The more stringent cutoff of ≥7 behaved similarly to the threshold of ≥5 in terms of predictors and may be utilized in situations in which resources are limited.

A single-item measure of sleep difficulties can flag patients for follow-up, but additional assessments are likely required to triage patients to appropriate care as individuals screening positive for sleep difficulties using a single item are inherently a heterogeneous population in terms of the type of sleep problems experienced, comorbidities, and specific drivers of sleep disturbance. Some sleep difficulties may be driven by symptoms such as pain and coughing, and these sleep difficulties may resolve when the inciting symptom is effectively medically managed. Some individuals may also experience transient disruptions in sleep quality related to the initial shock of diagnosis and treatment initiation that improve without behavioral treatment once patients acclimate to a new diagnosis and treatment regimen. However, certain disturbances (e.g., insomnia) are unlikely to resolve in the absence of treatment. For a significant proportion of survivors experiencing sleep disturbances at diagnosis, these issues become chronic if left untreated due to behavioral adaptations that perpetuate nocturnal restlessness and insomnia (2, 5). Although core interventions such as cognitive behavioral therapy for insomnia (CBT-I) and pharmacologic treatments where appropriate are effective for both cancer survivors (43, 50) and the general population, oncology patients may also benefit from tailored approaches that address cancer-specific factors (e.g., management of comorbid symptoms such as fatigue, pain, and psychologic distress). Integrated care models that address multiple symptoms simultaneously may be particularly effective for cancer survivors. Future research should explore drivers of sleep difficulties and trajectories of sleep problems among individuals screening positive using a single item to inform the development of a care pathway.

Given the strong overlap between sleep difficulties and symptoms of distress, anxiety, and depression, a practical approach may be to follow up on sleep concerns among individuals who already screen positive for these related issues (37). At our cancer center, we developed automated referral pathways for distress, malnutrition, anxiety, and depression (28, 51). Leveraging such existing systems to incorporate sleep-related follow-up could enhance efficiency and reduce missed opportunities for intervention. This integrated strategy may be especially valuable in high-volume clinical settings where adding new screening items or workflows is challenging. However, it is unknown how many patients with significant and treatable sleep issues may be missed using this strategy.

Results of this study must be interpreted within the context of its limitations, notably its retrospective exploratory design and reliance on a single item or brief measures of multidimensional constructs (e.g., sleep difficulties, pain, anxiety, depression, distress, problems in relationships, financial strain). Additionally, although the sample is large and diverse, it reflects patients treated at academic-community cancer center sites in the southeastern United States, which may limit generalizability. As analyses were exploratory in nature, we did not account for multiplicity; results should therefore be interpreted with caution given potential type I error inflation. Another limitation of this study is the exclusion of patients with multiple primary cancers. Although this approach was chosen to enhance analytic clarity, it also excludes a clinically relevant subgroup of survivors who may be at heightened risk for sleep disturbances due to cumulative disease and treatment burden. Future research should examine sleep outcomes specifically among individuals with multiple primaries to better characterize their unique survivorship needs.

Although most patients in this sample (>75%) were newly diagnosed (within ± 90 days of diagnosis), some may have been switching treatments or returning after progression, explaining the variability in the timing of patients’ initial EDS with respect to their diagnosis. However, sensitivity analyses limiting the sample to those within 90 days of diagnosis show consistency in results with respect to sleep disturbance prevalence and correlates, demonstrating that sleep disturbances are prevalent early in treatment, potentially before cumulative effects are experienced. Although the heterogeneity in the timing of patients’ initial EDS with respect to their diagnosis and treatment limits our ability to understand the relationship between these factors and sleep, these findings highlight that sleep difficulties are common even at the earliest stages of the cancer journey. Sleep difficulties may persist or intensify as treatment and disease burden accumulate over time (2, 5), underscoring the need for early identification and intervention to prevent chronicity.

Taken together, our findings are consistent with the literature highlighting that sleep difficulties are prevalent, interconnected, yet underaddressed in oncology settings. Our findings underscore the need for systematic screening and tailored interventions for sleep difficulties in oncology. Given the strong associations with distress and pain, integrated care models that address multiple symptoms simultaneously may be particularly effective. Future research should explore longitudinal trajectories of sleep disturbances and differences by cancer type and develop and test scalable interventions, such as brief behavioral therapies (e.g., CBT-I), digital health tools, and care pathways that leverage routine screening data to identify and refer patients at risk. Additionally, implementation science studies are needed to evaluate the effectiveness of automated referral systems and multisymptom management strategies within routine oncology workflows.

### Conclusions

In this large and diverse outpatient oncology population, sleep difficulties were common, with more than one third reporting potentially clinically significant symptoms. Sleep difficulties co-occurred with pain, distress, anxiety, and depression, underscoring the value of integrated, multisymptom approaches to management. Systematic screening and targeted follow-up could improve identification and care for oncology patients, who are at high risk for sleep disturbance due to multiple co-occurring issues. Future research should clarify sleep disturbance trajectories throughout treatment and test scalable interventions within routine oncology workflows.

## Data Availability

The data and code that support the findings of this study are available from the corresponding author upon reasonable request.
